# Supplementary material for: In vivo18F-DOPA PET imaging identifies a dopaminergic deficit in a rat model with a G51D α-synuclein mutation
Source: Front Neurosci. 2023 May 24;17:1095761. doi: 10.3389/fnins.2023.1095761 (PMC10244711; doi:10.3389/fnins.2023.1095761)
Supplement: Supplementary file 1 [file Data_Sheet_1.docx]

**SUPPLEMENTARY INFORMATION FOR**

***In vivo* ^18^F-DOPA PET imaging identifies a dopaminergic deficit in a rat model with a G51D α-synuclein mutation**

Victoria Morley^1^, Karamjit Singh Dolt^1^, Carlos J. Alcaide-Corral^3^, Tashfeen Walton^3^, Christophe Lucatelli^3^, Tomoji Mashimo^2^, Adriana A. S. Tavares^3*^, Tilo Kunath^1*^

^1^Centre for Regenerative Medicine, Institute for Regeneration and Repair, School of Biological Sciences, The University of Edinburgh, 5 Little France Drive, Edinburgh, EH16 4UU, UK

^2^Division of Animal Genetics, Laboratory Animal Research Center, Institute of Medical Science, The University of Tokyo, 4-6-1 Shirokanedai, Minato-ku, Tokyo 108-8639

^3^University/BHF Centre for Cardiovascular Science, The Queen’s Medical Research Institute, The University of Edinburgh, 47 Little France Crescent, Edinburgh, EH16 4TJ, UK


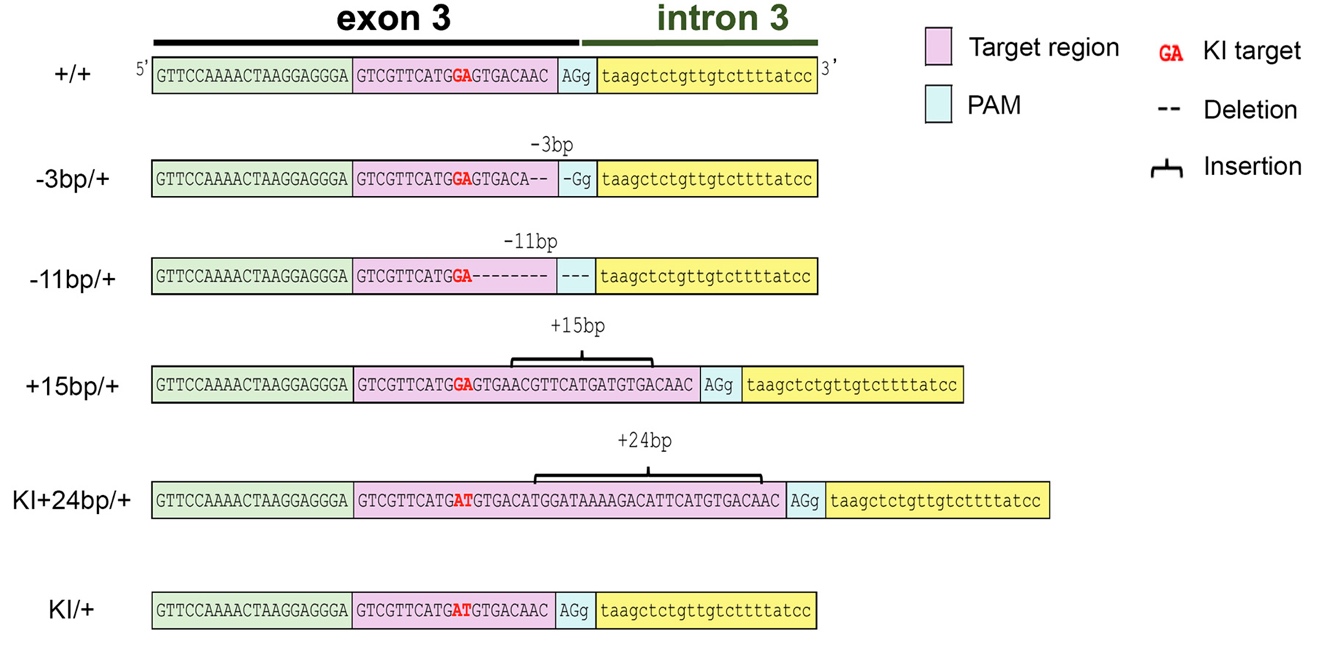


**Supplementary Figure S1. DNA sequence of CRISPR/Cas9 founder rats.** Five (5) of 11 F0 rats had mutations in the rat *SNCA* locus. Only one founder animal had the correct knock-in (KI) target with the GA to AT mutation without additional mutations.


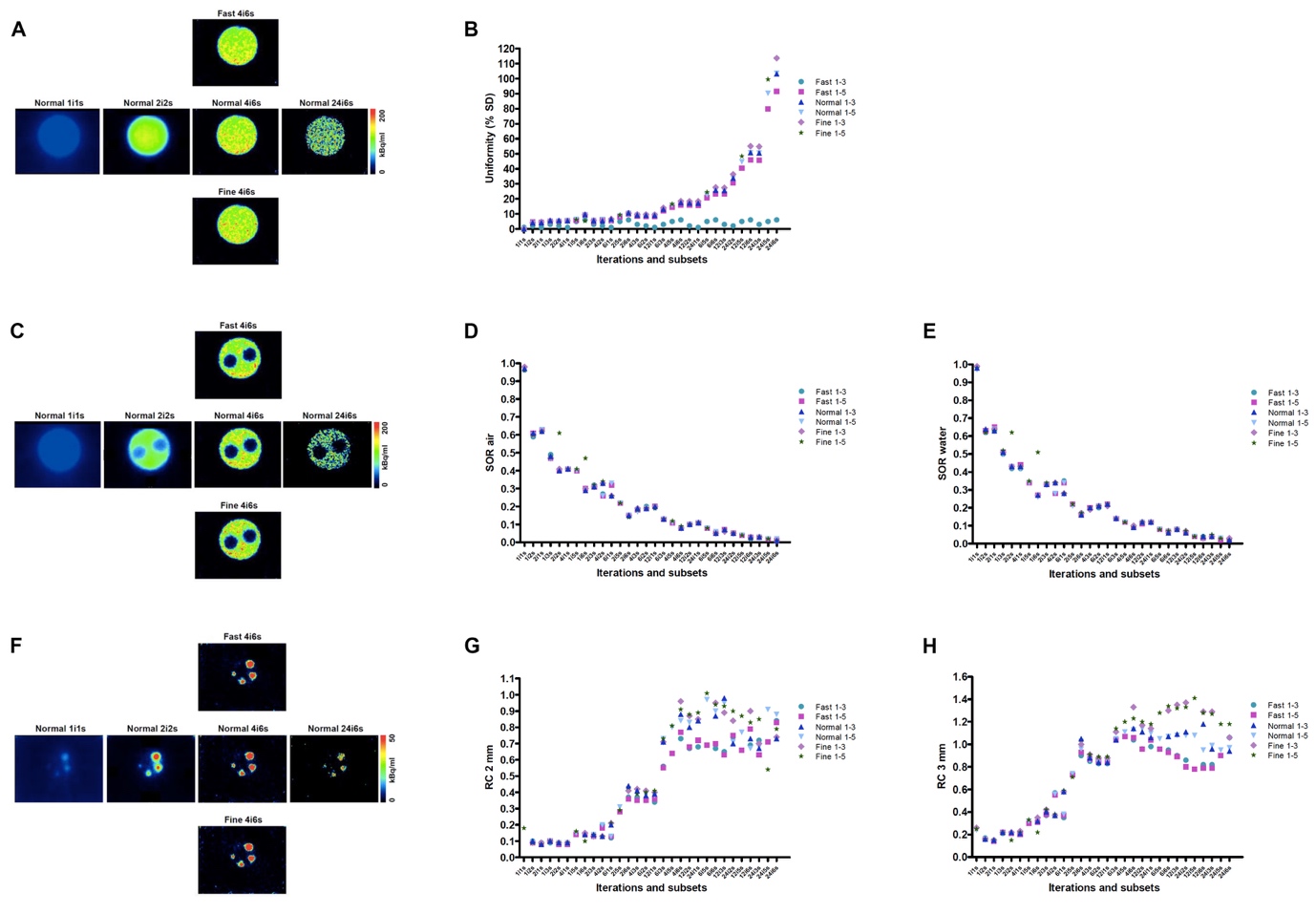


**Supplementary Figure S2. PET IQ Phantom data.** The phantom was filled

with 3.8 MBq of ^18^F-FDG solution. (A) Images of the central chamber after reconstructing the PET data using different methods varying the resolution (fast/normal/fine) and the number of iterations and subsets used for reconstruction. (B) Effect of the reconstruction method on the % standard deviation (% SD) in image uniformity. (C) Images of air- and water-filled inserts of the phantom. (D,E) Effect of spillover ratio (SOR) of activity into air (D) and water (E). (F) Images of rods of differing diameters (1mm – 5mm) in the phantom. (G,H) Recovery coefficients (RC) for 2 mm rods (G), and 3 mm rods (H).

**Supplementary Table 1.** Raw data and statistical tests

**For Figure 4B, 4C, 4D:** Ki, DVR, and EDVR data for each individual rat

| **Rat ID** | **Sex** | **Age (mon)** | **Genotype** | **Ki** | **DVR** | **EDVR** |
| --- | --- | --- | --- | --- | --- | --- |
| 16A0804B | Female | 5 | WT | 0.0080 | 1.470 | 0.459 |
| 16A0929A | Female | 5 | WT | 0.0090 | 1.570 | 0.563 |
| 16A0803B | Male | 5 | WT | 0.0100 | 1.360 | 0.335 |
| 16A0804C | Male | 5 | WT | 0.0140 | 1.460 | 0.440 |
| 16A0803A | Female | 5 | G51D/+ | 0.0070 | 1.410 | 0.397 |
| 16A1114B | Male | 5 | G51D/+ | 0.0090 | 1.490 | 0.471 |
| 16A0606C | Male | 5 | G51D/+ | 0.0070 | 1.390 | 0.352 |
| 16A0615C | Male | 5 | G51D/+ | 0.0050 | 1.410 | 0.403 |
| 16A0822A | Female | 5 | G51D/G51D | 0.0080 | 1.420 | 0.401 |
| 16A0928A | Female | 5 | G51D/G51D | 0.0070 | 1.440 | 0.425 |
| 16A0606B | Male | 5 | G51D/G51D | 0.0080 | 1.400 | 0.370 |
| 16A0615B | Male | 5 | G51D/G51D | 0.0070 | 1.510 | 0.490 |
| 16A0415A | Female | 11 | WT | 0.0080 | 1.440 | 0.416 |
| 16A0415B | Female | 11 | WT | 0.0150 | 1.410 | 0.413 |
| 16A0502A | Male | 11 | WT | 0.0050 | 1.400 | 0.377 |
| 16A0516A | Male | 11 | WT | 0.0069 | 1.450 | 0.428 |
| 16A0421A | Female | 11 | G51D/+ | 0.0150 | 1.620 | 0.670 |
| 16A0421B | Female | 11 | G51D/+ | 0.0090 | 1.440 | 0.416 |
| 16A0421C | Female | 11 | G51D/+ | 0.0100 | 1.400 | 0.365 |
| 16A0502B | Male | 11 | G51D/+ | 0.0130 | 1.520 | 0.551 |
| 16A1114A | Female | 11 | G51D/G51D | 0.0090 | 1.510 | 0.499 |
| 16A1102B | Male | 11 | G51D/G51D | 0.0080 | 1.550 | 0.535 |
| 16A1115A | Male | 11 | G51D/G51D | 0.0070 | 1.520 | 0.502 |
| 16A1115B | Male | 11 | G51D/G51D | 0.0060 | 1.480 | 0.464 |
| 16A0929B | Female | 16 | WT | 0.0100 | 1.610 | 0.564 |
| 16A1102A | Female | 16 | WT | 0.0165 | 1.540 | 0.535 |
| 16A0928B | Male | 16 | WT | 0.0090 | 1.610 | 0.592 |
| 16A1020C | Male | 16 | WT | 0.0079 | 1.390 | 0.381 |
| 16A0822B | Female | 16 | G51D/+ | 0.0110 | 1.470 | 0.465 |
| 16A1020A | Female | 16 | G51D/+ | 0.0065 | 1.430 | 0.418 |
| 16A0822C | Male | 16 | G51D/+ | 0.0080 | 1.340 | 0.317 |
| 16A1020B | Male | 16 | G51D/+ | 0.0076 | 1.430 | 0.410 |
| 17A0410A | Female | 16 | G51D/G51D | 0.0065 | 1.407 | 0.337 |
| 17A0410B | Male | 16 | G51D/G51D | 0.0070 | 1.410 | 0.393 |
| 17A0412A | Male | 16 | G51D/G51D | 0.0070 | 1.320 | 0.303 |
| 17A0412B | Male | 16 | G51D/G51D | 0.0080 | 1.400 | 0.375 |

**Statistical test for Ki:** one-way ANOVA with Tukey’s multiple comparisons

| **Comparison** | **P value** | **Significance** |
| --- | --- | --- |
| 5m WT vs. 5m G51D/+ | 0.075 | ns |
| 5m WT vs. 5m G51D/G51D | 0.136 | ns |
| 5m G51D/+ vs. 5m G51D/G51D | 0.900 | ns |
| 11m WT vs. 11m G51D/+ | 0.385 | ns |
| 11m WT vs. 11m G51D/G51D | 0.832 | ns |
| 11m G51D/+ vs. 11m G51D/G51D | 0.178 | ns |
| 16m WT vs. 16m G51D/+ | 0.360 | ns |
| 16m WT vs. 16m G51D/G51D | 0.146 | ns |
| 16m G51D/+ vs. 16m G51D/G51D | 0.787 | ns |

**Statistical test for DVR:** one-way ANOVA with Tukey’s multiple comparisons

| **Comparison** | **P value** | **Significance** |
| --- | --- | --- |
| 5m WT vs. 5m G51D/+ | 0.643 | ns |
| 5m WT vs. 5m G51D/G51D | 0.861 | ns |
| 5m G51D/+ vs. 5m G51D/G51D | 0.900 | ns |
| 11m WT vs. 11m G51D/+ | 0.276 | ns |
| 11m WT vs. 11m G51D/G51D | 0.141 | ns |
| 11m G51D/+ vs. 11m G51D/G51D | 0.883 | ns |
| 16m WT vs. 16m G51D/+ | 0.099 | ns |
| 16m WT vs. 16m G51D/G51D | 0.036 | * p < 0.05 |
| 16m G51D/+ vs. 16m G51D/G51D | 0.784 | ns |

**Statistical test for EDVR:** one-way ANOVA with Tukey’s multiple comparisons

| **Comparison** | **P value** | **Significance** |
| --- | --- | --- |
| 5m WT vs. 5m G51D/+ | 0.643 | ns |
| 5m WT vs. 5m G51D/G51D | 0.823 | ns |
| 5m G51D/+ vs. 5m G51D/G51D | 0.900 | ns |
| 11m WT vs. 11m G51D/+ | 0.301 | ns |
| 11m WT vs. 11m G51D/G51D | 0.304 | ns |
| 11m G51D/+ vs. 11m G51D/G51D | 0.900 | ns |
| 16m WT vs. 16m G51D/+ | 0.097 | ns |
| 16m WT vs. 16m G51D/G51D | 0.019 | * p < 0.05 |
| 16m G51D/+ vs. 16m G51D/G51D | 0.575 | ns |

**Supplementary Table 2.** Raw data and statistical tests

**For Figure 4E:** Left and right striatal EDVR data for each individual rat

| **Rat ID** | **Sex** | **Age (mon)** | **Genotype** | **Left Striatum** | **Right Striatum** |
| --- | --- | --- | --- | --- | --- |
| 16A0804B | Female | 5 | WT | 0.042 | -0.043 |
| 16A0929A | Female | 5 | WT | 0.072 | -0.078 |
| 16A0803B | Male | 5 | WT | 0.034 | -0.035 |
| 16A0804C | Male | 5 | WT | -0.143 | 0.125 |
| 16A0803A | Female | 5 | G51D/+ | -0.05 | 0.05 |
| 16A1114B | Male | 5 | G51D/+ | -0.11 | 0.1 |
| 16A0606C | Male | 5 | G51D/+ | 0.1 | -0.11 |
| 16A0615C | Male | 5 | G51D/+ | -0.23 | 0.19 |
| 16A0822A | Female | 5 | G51D/G51D | -0.15 | 0.13 |
| 16A0928A | Female | 5 | G51D/G51D | -0.04 | 0.04 |
| 16A0606B | Male | 5 | G51D/G51D | -0.16 | 0.14 |
| 16A0615B | Male | 5 | G51D/G51D | -0.08 | 0.07 |
| 16A0415A | Female | 11 | WT | -0.02 | 0.02 |
| 16A0415B | Female | 11 | WT | 0.06 | -0.06 |
| 16A0502A | Male | 11 | WT | -0.09 | 0.08 |
| 16A0516A | Male | 11 | WT | -0.19 | 0.16 |
| 16A0421A | Female | 11 | G51D/+ | 0.09 | -0.09 |
| 16A0421B | Female | 11 | G51D/+ | -0.21 | 0.18 |
| 16A0421C | Female | 11 | G51D/+ | -0.09 | 0.08 |
| 16A0502B | Male | 11 | G51D/+ | -0.37 | 0.27 |
| 16A1114A | Female | 11 | G51D/G51D | 0.04 | -0.04 |
| 16A1102B | Male | 11 | G51D/G51D | -0.09 | 0.08 |
| 16A1115A | Male | 11 | G51D/G51D | -0.11 | 0.1 |
| 16A1115B | Male | 11 | G51D/G51D | -0.12 | 0.1 |
| 16A0929B | Female | 16 | WT | -0.091 | 0.083 |
| 16A1102A | Female | 16 | WT | 0.007 | -0.007 |
| 16A0928B | Male | 16 | WT | -0.029 | 0.028 |
| 16A1020C | Male | 16 | WT | -0.238 | 0.193 |
| 16A0822B | Female | 16 | G51D/+ | 0 | 0 |
| 16A1020A | Female | 16 | G51D/+ | -0.2 | 0.17 |
| 16A0822C | Male | 16 | G51D/+ | 0.08 | -0.09 |
| 16A1020B | Male | 16 | G51D/+ | -0.05 | 0.05 |
| 17A0410A | Female | 16 | G51D/G51D | 0.29 | -0.41 |
| 17A0410B | Male | 16 | G51D/G51D | 0.16 | -0.19 |
| 17A0412A | Male | 16 | G51D/G51D | 0.13 | -0.15 |
| 17A0412B | Male | 16 | G51D/G51D | 0.11 | -0.12 |

**Statistical test:** Paired Student’s *t*-test

| **Comparison** | **P value** | **Significance** |
| --- | --- | --- |
| 5m WT L vs R striatum | 0.930 | ns |
| 5m G51D/+ L vs R striatum | 0.395 | ns |
| 5m G51D/G51D L vs R striatum | 0.031 | * p < 0.05 |
| 10m WT L vs R striatum | 0.350 | ns |
| 10m G51D/+ L vs R striatum | 0.239 | ns |
| 10m G51D/G51D L vs R striatum | 0.165 | ns |
| 16m WT L vs R striatum | 0.196 | ns |
| 16m G51D/+ L vs R striatum | 0.554 | ns |
| 16m G51D/G51D L vs R striatum | 0.035 | * p < 0.05 |
